# Mucosal Dynamics Contributing to Innate Immune Responses to HIV in the Human Female Genital Tract

**DOI:** 10.3390/v18050542

**Published:** 2026-05-08

**Authors:** Genna E. Moldovan, Gabriel P. Faber, Marta Rodriguez-Garcia

**Affiliations:** 1 Department of Obstetrics and Gynecology, School of Medicine, Wayne State University, Detroit, MI 48201, USA; gmoldovan@wayne.edu (G.E.M.); id6136@wayne.edu (G.P.F.); 2 C.S. Mott Center for Human Growth and Development, School of Medicine, Wayne State University, Detroit, MI 48201, USA; 3 Department of Biochemistry, Microbiology and Immunology, School of Medicine, Wayne State University, Detroit, MI 48201, USA

**Keywords:** HIV, female genital mucosa, innate immunity, menopause, dendritic cells, neutrophils, innate lymphoid cells

## Abstract

HIV is primarily acquired in women at the female genital mucosa through heterosexual contact. Mucosal immune cells reside adjacent to, within, below, and distant from the epithelium that lines the surface of the female genital tract (FGT) mucosa. Innate immune cells play dual roles in HIV acquisition, both poised to rapidly recognize and respond to HIV, but are also capable of promoting HIV infection locally and distantly in the lymph nodes. In this review we emphasize recent human research on the roles of specific innate immune cells in HIV pathogenesis in the FGT, including dendritic cells, macrophages, neutrophils and innate lymphoid cells. We review how FGT mucosal dynamics, including anatomical compartmentalization, menstrual cycle regulation, reproductive history, menopause and chronological aging contribute to tissue conditioning of these cells and changes in HIV susceptibility in women throughout their lives.

## 1. Introduction

### 1.1. HIV Pathogenesis in the Female Genital Tract

The female genital tract (FGT) is a primary site of initiation of HIV infection in women. Epidemiological surveillance shows that heterosexual transmission is the main mechanism for HIV acquisition, and *in vivo* animal studies and *ex vivo* human studies have detected HIV infection at multiple anatomical sites within the FGT including the vagina, cervix, endometrium, fallopian tubes and ovaries [1,2,3,4,5,6,7,8,9]. The FGT is designed for reproduction, a task supported by the mucosal immune system. The immune system in the FGT is specifically shaped in a compartmentalized manner to aid in different events needed for reproduction including interaction with the allogeneic sperm, embryo implantation and pregnancy, and pre- and post-pregnancy tissue conditioning and remodeling [10,11]. Importantly, the same immune cells that reside in the FGT performing reproductive functions are the ones that can encounter HIV, which, as a sexually transmitted pathogen, will be carried into the FGT via semen.

The epithelium, mucus, and luminal secretions provide the first barrier of protection against HIV. The upper FGT, including the fallopian tubes, endometrium, and endocervix, is lined with simple columnar epithelium linked by tight junctions that provide what is considered a relatively weak layer of protection due to the physical monolayer structure [12,13], and at the same time can respond to HIV, initiating first-line innate immune responses [14,15]. The lower FGT, including the ectocervix and vagina, is lined with stratified squamous epithelium that provides protection through multiple epithelial layers and continuous sloughing of the upper layer of epithelium, carrying away any potential pathogens [12,13,16,17]. Epithelial cell direct responses to HIV are a critical component of innate protection and their contribution to HIV pathogenesis has been extensively reviewed elsewhere (see [2,16,18,19,20,21]). HIV-1 surpasses these epithelial barriers through different mechanisms [22,23] and establishes infection in the target immune cells located within the epithelial and subepithelial compartments, with CD4+ T cells most efficiently supporting viral replication [3,7,8,24,25,26]. However, virions will also encounter a variety of innate immune cells poised to immediately respond to incoming pathogens [1,2]. These pre-existing physical, chemical and immunological barriers can prevent infection in most cases, with male-to-female transmission rates per heterosexual act estimated around 0.08% [27]. However, conditions in which the epithelial barriers are weakened or disrupted or in which the availability of HIV-target cells is increased, whether physiological or pathological, will facilitate HIV-1 access to immune cells within the FGT [2]. While the events that lead to the establishment of HIV infection in the genital mucosa are being intensively studied and reviewed (primarily mechanisms that induce genital inflammation, compromise epithelial barriers and allow HIV-target cell infection) [16,20,21,23,28,29,30,31,32], the antiviral responses triggered after mucosal HIV exposure from innate immune cells that are not HIV-target cells are less understood.

### 1.2. Dynamic Changes in the Genital Mucosa

Different from other mucosal surfaces, the FGT mucosa undergoes dramatic dynamic changes throughout a woman’s lifespan. The immune system and the tissue environment in the FGT are tightly controlled by sex hormones that dictate cyclical monthly remodeling and regulation during reproductive years to optimize conditions for implantation [10]. At the end of the reproductive years, sex hormone decline drives menopausal changes associated with the loss of reproductive potential. These dynamic changes regulate local inflammation, immune cells, and genital microbiome composition and have consequences for susceptibility to genital infections, including HIV infection [33,34,35,36,37,38,39]. Variations in susceptibility to HIV infection through the menstrual cycle have been described [40,41,42], supporting the existence of a window of vulnerability for HIV acquisition during reproductive years [43]. Further, following menopause, histological and immunological changes in the FGT lead to increased biological susceptibility to infection, as evidenced by epidemiological and *in vitro* studies [7,44,45,46,47].

Immune cell distribution and the mucosal events of HIV pathogenesis in the female genital tract have been recently reviewed elsewhere [1,2,10,16,48]. In this review we will focus on the initial interactions between HIV and specific mucosal innate immune cell populations relevant for HIV pathogenesis and recently demonstrated to rapidly respond to HIV exposure including dendritic cells, neutrophils and innate lymphoid cells. We will discuss how these interactions and susceptibility to HIV infection dynamically change throughout women’s lifespan with a special emphasis on recent literature and human research.

## 2. Tissue Compartmentalization of Innate Immune Cell Distribution and HIV Responses

### 2.1. Mononuclear Phagocyte Populations

The different anatomical compartments of the FGT harbor multiple innate immune cell populations that display subset differences depending on location. Perhaps the most well-studied in the FGT is the tissue-specific modification of human mononuclear phagocytes (MNPs) (reviewed extensively in [49]). MNPs include dendritic cells (DCs), Langerhans cells (LCs), macrophages and monocyte-derived cells, all of which are found throughout the upper and lower FGT in a compartmentalized manner [2,16,50,51,52,53,54,55]. Within the FGT, specific DC subsets are present in the epidermis, epithelial layers, subepithelial space and deeper within the tissue. Epidermal DCs found within the external female genitalia skin, including the labia majora and labia minora, are typically LCs in healthy individuals where they reside in between keratinocyte tight junctions but still maintain barrier integrity [49,56,57,58]. Vaginal epithelial DCs are characterized as Langerin+ CD1a+ and DC-SIGN- [58]. Conventional CD11c+ DCs in the FGT constitute less than 10% of CD11c+ CD11b- DCs present in the endometrium, and less than 5% in the endocervix and ectocervical tissues [50]. Different subsets of conventional DCs (cDC1, cDC2 and cDC3) have been described throughout the FGT. cDC1s are characterized by XCR1 expression, high CD141 and low CD11c expression [52]. cDC1s are rare in the FGT and seem to be enriched in the endometrium, where they can also be identified by expression of CD103 [49,50,51,59]. cDC2s are present throughout the FGT and express CD1a, CD1c, CD11c and SIRPa and differ from circulating cDC2s in that they present with a more activated phenotype, represented by high expression of CD80, CD83, and CD86 [49,50]. Finally, recent work has shown that CD14+ DCs represent a heterogeneous population of DCs that include DC3s, characterized as CD14+ CD1c+ cells [50,51,60], monocyte-derived DCs and CD14+ CD1c- monocytic-like cells [51]. Investigation of the distribution and differential function of MNP subsets within the FGT is ongoing. More research is needed to understand the specific contribution of different mononuclear phagocyte populations to HIV acquisition, particularly as it relates to macrophages.

The distribution and characteristics of MNPs in the FGT are highly relevant since these cells closely interact with the epithelium, are specialized for tissue surveillance and antigen presentation, and are likely to encounter HIV early upon exposure. CD14-expressing DCs display enhanced viral capture ability early after exposure [50,52,61,62]. Additionally, CD14+ DCs and cDC2 from the FGT were shown to rapidly respond to HIV stimulus (within 30 min) with significant transcriptional increases in cytokine signaling genes and anti-viral genes [51]. Indeed, CD14+ and CD1a+ DCs also rapidly secreted cytokines and chemokines in response to HIV stimulus in vitro, many of which have antiviral properties [50,51], and released specific antimicrobials with direct anti-HIV activity, including Elafin and SLPI, which also display anti-inflammatory properties [50,63,64]. MNPs represent a first-line response to mucosal HIV exposure due to their location and tissue-specific expression and function.

### 2.2. Neutrophils

Like MNPs, neutrophils are also found throughout the upper and lower FGT, where they are relatively abundant, representing 5–30% of immune cells [65,66,67,68,69]. Neutrophils are also abundant on the surface of the endocervix and can be recovered with an endocervical cytobrush or cervicovaginal lavage [70,71]. However, genital neutrophils remain understudied and whether there are different populations or functional differences based on location remains to be elucidated. Evidence for tissue environment reprogramming of neutrophils has been demonstrated in murine models in other organ systems [72,73,74,75,76], but has yet to be defined in the human FGT and in the context of HIV infection. Neutrophils from blood and the FGT directly respond to HIV with the release of neutrophil extracellular traps (NETs), which immobilize and inactivate HIV, preventing subsequent infection of target cells [65,66,77,78,79]. The ability to rapidly respond to and inactivate HIV places neutrophils as potential first-responder effector cells during mucosal HIV exposure.

However, neutrophils can also contribute to tissue inflammation, known to be a critical driving factor for HIV susceptibility [30,80,81,82,83,84]. A recent animal study evaluated the interaction between neutrophils and bacterial vaginosis [85]. This study found that neutrophil presence in the vagina under homeostatic conditions did not alter epithelial integrity; however, when bacterial vaginosis was present, neutrophils were persistently activated and their exacerbated responses resulted in epithelial disruption [85]. These findings provide proof of concept that neutrophils can have both protective and deleterious effects depending on the mucosal inflammatory context, with important implications for HIV acquisition.

### 2.3. Innate Lymphoid Cells

Innate lymphoid cells (ILCs) represent another understudied population of innate immune cells in the FGT. Little is known about ILC distribution and tissue-specific function in the human FGT, despite their key presence in many mucosal tissues [86]. Within the human FGT, ILCs were initially identified in the endometrium, comprising predominantly ILC3 subpopulations [87]. More recently, the distribution of ILCs throughout the FGT was characterized, showing that ILCs are more abundant in the endometrium compared to the endocervix and ectocervix. In addition, subset distribution differed by tissue with ILC1s (CD127+ CD294− CD117−) more abundant in the ectocervix, and ILC3s (CD127+ CD117+) more abundant in the endometrium, while ILC2s (CD127+ CD294+) were minimally present in the FGT [88]. Functional and spatial differences have also been identified. CD161 expression on ILC1s and ILC3s was reduced in the endometrium compared to the endocervix and ectocervix and was identified as a marker of cytokine production [88]. In contrast, ILC degranulation (measured as CD107a expression) was limited to NKp44+ ILC3s and significantly enhanced in the endometrium [88]. This indicates very fine-tuning of ILC function in the human FGT depending on anatomical location. Importantly, genital ILCs were able to respond to HIV stimulation very rapidly with transcriptional changes, degranulation and cytokine production (IFNγ and IL-22), suggesting a potential role for ILCs in the initial response to mucosal HIV exposure [88]. Mouse ILCs indeed possess anti-viral capacity (reviewed extensively in [86,89]), but direct antiviral activity of human ILCs remains to be determined. IL-22 and IFN-γ promote antiviral tissue states, suggesting that ILCs may contribute to the initial events of mucosal HIV acquisition. The investigation of ILC roles in HIV pathogenesis in the FGT remains an open area of scientific exploration.

## 3. Mechanisms for HIV Recognition, Capture and Internalization by Innate Immune Cells in the FGT

**Figure 1:**
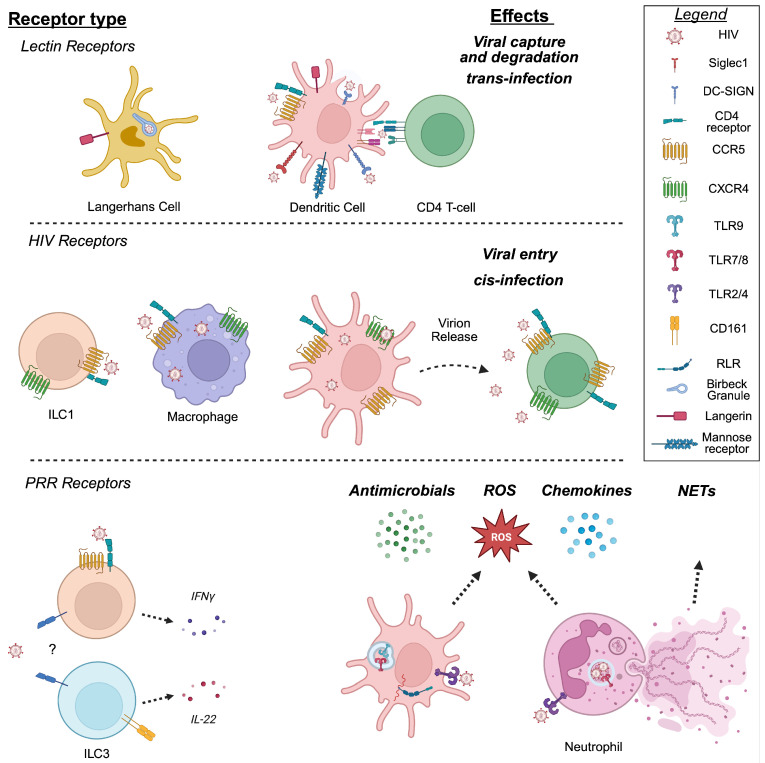
A summary of HIV recognition and responses by innate immune cells of the FGT. HIV sensing, recognition, and entry are mediated by C-type lectin receptors (**top panel**), HIV coreceptors CCR5, CXCR4 and CD4 (middle panel), and pattern recognition receptors (**bottom panel**). **Top Panel**: The lectin receptors on dendritic cells bind HIV virions and mediate internalization for degradation in Birbeck granules (Langerhans Cells) or HIV capture and viral transfer to CD4+ T cells in a process called trans-infection (Siglec1, DC-SIGN, Mannose receptor). Trans-infection can occur locally or after migration to lymph nodes. **Middle Panel**: The HIV co-receptors, CD4, CXCR4, and CCR5, enable viral entry and infection in target cells including CD4+ T cells, macrophages, and dendritic cells. ILC1s express HIV receptors but whether they support productive infection is unknown. **Bottom Panel**: Pattern recognition receptors that recognize HIV and induce anti-HIV responses including antimicrobials, ROS, and chemokine secretions are shown. It is not currently known which receptors mediate HIV sensing by FGT ILCs denoted by ?, but their cytokine responses to HIV exposure are shown.

Mechanisms specialized in innate sensing and capture of pathogens play critical roles during the initial events of mucosal HIV exposure. These cell–HIV interactions are mediated by an array of innate receptors called pathogen recognition receptors (PRRs). PRRs that recognize HIV and have been studied in innate immune cells from the FGT include Toll-like receptors (TLRs) and RIG-I-like receptors (RLRs), which recognize specific viral components and activate innate immune responses, and C-type lectin receptors (CLRs), which bind glycans present on the viral envelope, mediating viral capture. HIV entry into innate immune cells can also be mediated by classical HIV receptors. Figure 1 summarizes interactions between HIV and innate immune cells.

### 3.1. Innate Responses Induced by Toll-like Receptor Stimulation

DCs can sense HIV and launch early antiviral responses through the secretion of cytokines, chemokines and antimicrobials [50,51]. HIV is an ssRNA retrovirus and DCs contain a variety of PRRs that recognize viral components including TLRs, expressed on the surface membrane and in endosomes, and RLRs that perform cytosolic detection [51,55,90]. In the lower FGT, LCs express TLRs 1-8 indicating the ability to sense HIV and other pathogenic insults [56]. Plasmacytoid dendritic cells use TLR7 to sense single-stranded RNA (ssRNA) viruses and do not require infection for viral sensing since TLR7 is in endosomes [91]. A recent study utilizing single-cell sequencing identified differential expression of TLRs in specific subsets of FGT DCs; CD14 DCs were enriched for *TLR4*, and *TLR2* associated with HIV binding [51]. Interestingly, the study identified a subset of nonclassical monocytes that expressed *TLR8*, previously shown as the main HIV sensing receptor in DCs in other systems [90], in addition to the gene encoding the RIGI receptor, *DDX58* [51]. While these receptors have been transcriptionally identified in FGT MNPs, functional confirmation of HIV binding in these MNP subsets of the FGT remains an open area of exploration.

Neutrophils express TLRs 1-10 except TLR3, and preferentially express TLR8 over 7 [65,92,93]. Neutrophils recognize HIV through TLR7 and TLR8 which results in the release of ROS and NETs [65,66,77]. Interestingly, in genital neutrophils, TLR8 and 7 are stimulated by HIV in a sequential manner, with TLR8 mediating the initial triggering of NETs, while TLR7 mediates NET maintenance and ROS production, likely through different intracellular signaling mechanisms [65]. Importantly, blood and genital neutrophils employ different mechanisms to sense and respond to HIV. While genital neutrophils rely primarily on TLR8 for early NET release, blood neutrophils can additionally sense the HIV envelope through a yet unknown mechanism to induce rapid intracellular calcium signaling and calcium-dependent NET release, which is independent of TLR8 stimulation [65].

### 3.2. Viral Capture and Trans-Infection Mediated by C-Type Lectin Receptors

In the FGT, CLRs described in HIV pathogenesis include langerin, DC-SIGN, Siglec-1 and the mannose receptor. Langerin (CD207) is expressed by LCs found in the lower reproductive tract in the interepithelial spaces [52,53,94,95] and also by subsets of CD1c+ and CD1a+ cDC2s throughout the FGT [50,52,58,59]. In LCs, langerin acts as an antiviral mechanism by mediating HIV binding and internalization into Birbeck granules, which degrade pathogens [96,97]. In contrast, langerin+ cDC2s lack Birbeck granules and support viral infection [52,58]. DC-SIGN (CD209) is expressed on many subsets of MNPs including CD14+ DCs, DC3s, monocyte-derived macrophages (MDMs), and macrophages that are most frequently found in the subepithelial spaces of the genital mucosa [50,51,53,95,98]. DC-SIGN binds to gp120 and can mediate the transfer of HIV to CD4+ T cells [99,100]. Although this initial work using *in vitro*-generated monocyte-derived DCs indicated that DC-SIGN was critical for viral capture, studies with primary DCs from the FGT found that DCs that lack DC-SIGN also capture HIV [50]. Siglec-1 (CD169) is another HIV-uptake receptor expressed on monocyte-derived DCs, MDMs, and cDC2s [53,101]. Siglec-1 enhances cell–cell contacts and propagation of HIV infection of adjacent cells and has been shown to participate in cervical capture and spread of HIV virions *in vitro* [61,102]. Recent work shows that activated DCs use actin machinery to rearrange Siglec-1 on the cell surface into distinct nanoclusters, which enhances HIV binding and capture [103]. This actin-mediated process also directs HIV into distinct virus-containing compartments (VCCs), which retain virions and promote trans-infection. The mannose receptor (CD206) is expressed on macrophages, MDMs, cDC2s, vaginal epithelial-associated DCs, and monocyte-derived DCs [104,105]. The mannose receptor can play two dichotomous roles: HIV internalization by endocytosis to be targeted for degradation [104], or membrane binding of HIV and transport to the lymph nodes, thereby propagating infection [105]. Although we review here a number of C-type lectin receptors that have been involved in interactions with HIV in the FGT, there are likely additional members of this family that also participate in MNP-HIV interactions. A recent study using single-cell sequencing described a large array of CLRs expressed on MNPs in a subset-specific manner in the FGT, many of which may be involved in the early viral recognition [51]. Experimental validation of predicted HIV-CLRs interactions in the FGT is needed.

Following viral binding by CLRs, HIV can be transmitted from DCs to CD4+ T cells through two major mechanisms termed trans- and cis-infection. Trans-infection is characterized by virions coating DC’s cell surface that then come into contact with CD4+ T cells in the early hours of infection (within 2–6 h) or virions are held in virus-containing compartments and released into the environment, leading to infection of adjacent cells [106,107]. This mechanism is considered a main contributor to early infection propagation within the local environment or the draining lymph nodes where DCs and LCs migrate to present antigens to CD4+ T cells [2,53,101]. However, trans-infection by FGT DCs has only been demonstrated *in vitro* and in *ex vivo* explant models [52,61,62]; therefore, the timing, location and conditions in which trans-infection takes place *in vivo* in the human FGT represent a gap in knowledge due to obvious limitations [8,62,86]. Of note, trans-infection does not require the direct infection of the MNP itself. The second mechanism, cis-infection, may occur wherein an MNP infected with HIV produces infectious virions that are then transported to the MNP’s filopodia and interact with CD4+ T cells and induce infection through a virological synapse [53,108]. In the FGT, epithelium-associated DCs including CD1a+ vaginal epithelial DCs [58] and LCs [57] have been reported to support viral replication, but in general, DCs are not considered as primary target cells for viral infection, partly due to SAMHD1 expression, which prevents HIV replication in DCs [58,109,110].

### 3.3. HIV Entry Mediated by Classical HIV Receptors and Non-Classical Mechanisms

Canonical HIV receptors and coreceptors include CD4, CCR5 and CXCR4 and allow viral entry and initiation of viral replication inside the cells. Although both CCR5 and CXCR4 are abundant in the FGT, likely due to potential roles in reproduction [111,112], only HIV strains using CCR5 as a coreceptor mediate initial infection in mucosal tissues [9]. Potential reasons for preferable sexual transmission of CCR5-tropic HIV include seminal plasma influences on CD4+ T cell expression of CD4 and CCR5 [113], preferential viral replication in CCR5^high^ CD4+ T cells, and the propagation of trans-infection from mucosal environment cells including fibroblasts [114]. However, the exact mechanisms and reasons for CCR5 preferential infection in the female genital mucosa remain a major gap in knowledge. The majority of MNPs in the FGT co-express CD4 and CCR5/CXCR4, representing potential targets for infection [50,51,52,58]. Neutrophils express CXCR4 and low levels of CCR5, but normally lack CD4 expression, and do not represent cell targets for viral replication [65]. However, neutrophils internalize HIV particles through endocytosis, which contributes to the signaling pathways that lead to NET-release [65].

ILC1s in the FGT co-express CD4 and CCR5 [88], and respond to HIV stimulation, partly mediated through engagement of classical HIV receptors (CD4/CCR5), but it remains unknown whether ILC1s can become productively infected in the FGT [88]. In contrast, ILC3s do not express CD4, but can also respond to HIV stimulation, suggesting that HIV recognition in ILC3s is not mediated by classical HIV receptors [88]. Whether HIV sensing by genital ILCs is mediated through PRRs or through tissue-mediated indirect mechanisms remains unsolved [88].

## 4. Control of Innate Immune Cells in the FGT by Sex Hormones and Menstrual Cycle

The FGT is continuously exposed to fluctuating levels of endogenous sex hormones throughout a woman’s life. During reproductive years, sex hormones control endometrial remodeling in preparation for embryo implantation and endometrial shedding during menstruation [115]. This cyclical regulatory process includes control of immune cell populations, resulting in varying presence or function of specific immune cells at different time-points during the menstrual cycle [10,116,117,118,119,120,121]. In the endometrium, total immune cells increase during the late secretory phase and into menstruation, to perform tissue remodeling tasks, but there are no cyclical hormonal effects on the frequency of lower FGT immune populations [10]. Increased immune recruitment in the latter half of the secretory phase is facilitated by cytokine, chemokine, and growth factor secretion from epithelial and stromal cells under the guidance of sex hormones [10].

Sex hormones including estrogens and progesterone have been shown to have profound effects on immune cell function, either directly or indirectly through effects on the tissue environment [10], including control of T cell populations and function [117,118,122]. Sex hormones have also shown direct modulation of HIV infection in CD4+ T cells and macrophages [123,124,125,126]. While hormonal influence on T cell function and susceptibility to HIV infection has been an important focus for research, hormonal effects on innate immune cell function and their responses to HIV are less understood. Treatment of endometrial DCs with 17β-estradiol (E2) prior to HIV stimulation reduced the secretion of Elafin, and SLPI, but not CCL5 (RANTES) [50]. In addition, production of alpha defensins 1-3, important antimicrobials secreted by DCs and pDCs in response to HIV [127], was reduced with E2 treatment but not with progesterone treatment *in vitro* [128]. In another *in vitro* model utilizing monocyte-derived DCs pre-treated with E2, DCs decreased their response to viral stimulus to an RNA virus, Newcastle Disease virus, and had a decreased costimulatory capacity on CD4 naïve T cells [129]. These studies overall suggest that E2 would dampen DC-mediated innate responses to viruses. However, with a few exceptions, most studies to date have primarily utilized blood-derived cells like PBMCs, monocyte-derived macrophages, and monocyte-derived DCs to determine the effects of sex hormones on immune cell susceptibility to HIV infection and responses to HIV. This area represents a significant gap in the field, given that FGT-derived MNPs are heterogeneous and already exposed to sex hormones, likely resulting in differing responses to *in vitro* hormonal stimulation. There is a need to better define how sex hormones control mucosal innate immune cells and their responses to HIV.

Neutrophils are recruited into the endometrium to participate in tissue remodeling during menstruation [116,130]. However, most of our knowledge on sex hormone control of FGT neutrophils is derived from mouse models [131,132]. Pathway analysis using cervicovaginal lavage from women in different stages of the menstrual cycle identified increased neutrophil recruitment and accumulation pathways on the cervicovaginal luminal surface during the secretory phase [133]. Another recent study using cynomolgus macaques (which undergo menstrual cycles similar to women) identified variations in neutrophil populations during the menstrual cycle and increases in a specific neutrophil population with higher CD62L expression during menstruation in cervicovaginal cytobrush samples [134]. Similar studies analyzing neutrophil populations in humans are lacking.

Endometrial ILCs are also modified by the menstrual cycle. A recent study described increases in CD127- ILC3 around the time of menstruation, indicating a potential role in tissue repair. In contrast, CD127+ ILC3s were more abundant after menstruation, suggesting roles in the regeneration of the endometrium [119]. How these hormonal changes may affect innate immune cell responses to HIV is unknown. Given the critical and ubiquitous nature of sex hormones within the FGT, understanding how hormonal changes affect innate immune cell homeostatic function and responses to HIV remains an essential area of open investigation.

## 5. Menopause and Chronological Aging Affect HIV Susceptibility in the FGT

Beyond the hormonal influence of the menstrual cycle, there are significant changes that occur in the FGT following menopause and due to chronological age. Unique to humans, women undergo menopause and remain in a postmenopausal state for decades of their lives. Age-dependent changes in the FGT regarding barrier protection, cytokines, PRR expression and distribution of overall immune cells have been reviewed extensively elsewhere and will not be reviewed in detail here [33,34]. Epidemiological analyses have shown an increased odds ratio of 3.9 in HIV acquisition risk in women of peri and postmenopausal age (≥45 years) [47], suggesting a need to better understand the mechanisms of acquisition that affect postmenopausal innate immune function in the FGT. In a cervical explant study of postmenopausal- vs. premenopausal-derived tissue, the authors noted increased HIV susceptibility in postmenopausal ectocervical explants compared to premenopausal explants that was attributed to early inflammatory signaling in the postmenopausal explants [46]. Another study also showed that antiviral activity, specifically anti-HIV activity, of postmenopausal-derived cervical lavage was significantly decreased compared to premenopausal samples, regardless of menstrual cycle phase or contraceptive use [135]. Secreted factors shown to have anti-HIV activity *in vitro*, β-Defensin 2, MIP3α, IL-6 and SLPI [63,64,136,137,138,139], were lower in the cervical-vaginal lavage of postmenopausal women compared to premenopausal, suggesting decreased protection against HIV in the lower FGT in postmenopausal compared to premenopausal women [35]. Inflammatory markers have also been described to increase in the cervicovaginal lumen with age [36]. These studies suggest dysregulation of innate immune mechanisms of protection after menopause.

Regarding DC function, endometrial DCs from older women display increased T cell proliferation capacity compared to DCs from younger women, increased induction of CD103 expression on CD8+ T cells, and increased cytokine production, indicating increased activity and proinflammatory profile between FGT DCs from older and younger women [140,141]). Proteomic analysis of cervicovaginal lavage identified increased myeloid cell migration and phagocytosis pathways with age, also indicating enhanced activation [36].

Few studies have investigated the effect of menopausal status and chronological aging on neutrophil distribution, function, and responses to HIV [65,66,78]. Neutrophil extracellular trap (NET) formation in response to HIV was impaired in postmenopausal neutrophils from blood and FGT, but neutrophil presence was not altered in the FGT when comparing pre vs. postmenopausal women, indicating a dysregulation of neutrophil antiviral function with age [65]. Interestingly, although NET release in response to HIV was dysregulated in both blood and genital neutrophils, the molecular mechanisms driving this impairment were different, with rapid calcium responses dampened in blood neutrophils, but TLR8 signaling preferentially compromised in FGT neutrophils [65]. Another study demonstrated that incubation of blood neutrophils from pre and postmenopausal women with short-chain fatty acids, bacterial metabolites known to modulate immune responses, resulted in reduced and delayed anti-HIV responses from neutrophils, preferentially in postmenopausal neutrophils [78], consistent with reduced responsiveness to pathogen stimulation in neutrophils from older women. Proteomic pathway analysis of cervicovaginal secretions also detected reductions in pathways related to neutrophils and reactive oxygen species [36], further supporting impairment of neutrophil function with age. The characterization of FGT neutrophils in pre- and postmenopausal women, and any potential alterations in HIV responsiveness, remain open areas of investigation.

The percentage of ILCs in the FGT is also altered with age. A recent study demonstrated decreased presence of total ILCs with age in the ectocervix specifically [88]. In contrast, in the endometrium, aging modified the ILC subset distribution. As women aged, ILC1s were reduced while ILC3s increased without alterations in overall ILC presence [88]. These alterations in ILC populations and distribution likely underline functional changes that warrant future investigation.

Overall, these studies indicate dysregulated innate immune responses and increased activation and inflammation, pointing to postmenopausal women as a significant at-risk group for contracting HIV through sexual transmission and warrant a specific focus on understanding the interplay of menopause, chronological aging, and innate immune responses to HIV in the aging FGT.

## 6. Reproductive History Affects Innate Immune Cell Distribution and Function and HIV Susceptibility

Recent work has shown that the risk of HIV acquisition in women during late pregnancy and the peripartum period is 3–4-fold higher compared to that of a nonpregnant period [142], but the mucosal events that lead to enhanced susceptibility during this interval remain unknown. Maternal HIV research has justifiably focused on perinatal transmission and keenly on the prevention of HIV transmission to the fetus or newborn child [143,144,145]. A recent study showed that pregnant women living with HIV have differential circulating cytokine profiles (including increased proinflammatory IP-10 and T-helper associated cytokines) compared to pregnant women without HIV infection, and that these cytokine profiles in pregnant women living with HIV were strongly associated with smaller babies per gestational age [146]. Mounting evidence indicates that pregnancy induces long-lasting changes in immune cell populations [147]. This remodeling of the immune landscape after pregnancy includes local populations in the endometrium. A recent study reported the presence and persistence of specific uterine macrophage subsets after pregnancy that improve subsequent pregnancy outcomes [148]. The distribution and phenotype of CD127+ ILCs in the human endometrium were also shown to correlate with the time since women’s last pregnancy, with recent pregnancies associated with lower presence of CD127+ ILCs and higher expression of CD161 [88]. CD127- ILC3s were also reported to increase after parturition [119]. The dynamics and function of specific innate immune cell subsets during human pregnancy, the postpartum periods, long-lasting reproductive immune remodeling, and their role in HIV acquisition are significantly lacking. Basic mechanistic research is needed in this area.

## 7. Conclusions

The mucosal immune system in the FGT is critical for successful reproduction and protection against infections, including HIV acquisition. During reproductive years, monthly fluctuations in sex hormones dramatically modify the FGT to optimize conditions for pregnancy. Following pregnancy, immune populations are modified with long-lasting effects. Once reproductive potential is lost, after menopause, the immune system in the FGT is further altered, since the main reproductive function is no longer needed, but protection against infections remains necessary. These reproduction-related dynamic changes alter the composition and function of innate immune cells that provide tissue remodeling tasks and first-line defense against genital pathogens, including HIV. Dendritic cells, neutrophils and ILCs have diverse roles under homeostatic conditions and are involved in mucosal protection and the initial responses to HIV exposure. Understanding how innate immune cells behave in the context of the dynamic changes in the FGT will help us understand first-line protection against HIV and develop preventive strategies customized for women throughout their lives.

## Data Availability

No data were generated in this manuscript.

## Abbreviations

The following abbreviations are used in this manuscript:

FGT	Female genital tract
HIV	Human immunodeficiency virus
MNP	Mononuclear phagocyte
ILC	Innate lymphoid cell
DC	Dendritic cell
TLR	Toll-like receptor
CLR	C-type lectin receptor
RLR	RIG-I-like receptor
